# Acoustic Sensing Analytics Applied to Speech in Reverberation Conditions

**DOI:** 10.3390/s21186320

**Published:** 2021-09-21

**Authors:** Piotr Odya, Jozef Kotus, Adam Kurowski, Bozena Kostek

**Affiliations:** 1 Department of Multimedia Systems, Faculty of Electronics, Telecommunications and Informatics, Gdansk University of Technology, 11/12 Narutowicza Street, 80-233 Gdansk, Poland; jozkotus@pg.edu.pl (J.K.); adakurow@pg.edu.pl (A.K.); 2 Audio Acoustics Laboratory, Faculty of Electronics, Telecommunications and Informatics, Gdansk University of Technology, 11/12 Narutowicza Street, 80-233 Gdansk, Poland

**Keywords:** acoustic sensing, reverberation, speech intelligibility, digital signal processing, time-scale modification, speech rate, STI

## Abstract

The paper aims to discuss a case study of sensing analytics and technology in acoustics when applied to reverberation conditions. Reverberation is one of the issues that makes speech in indoor spaces challenging to understand. This problem is particularly critical in large spaces with few absorbing or diffusing surfaces. One of the natural remedies to improve speech intelligibility in such conditions may be achieved through speaking slowly. It is possible to use algorithms that reduce the rate of speech (RoS) in real time. Therefore, the study aims to find recommended values of RoS in the context of STI (speech transmission index) in different acoustic environments. In the experiments, speech intelligibility for six impulse responses recorded in spaces with different STIs is investigated using a sentence test (for the Polish language). Fifteen subjects with normal hearing participated in these tests. The results of the analytical analysis enabled us to propose a curve specifying the maximum RoS values translating into understandable speech under given acoustic conditions. This curve can be used in speech processing control technology as well as compressive reverse acoustic sensing.

## 1. Introduction

It is noteworthy to first introduce the notion of compressive sensing applied to acoustics, understood as reconstructing acoustic fields from a series of audio signal measurements [1,2,3,4]. Taking into account the sparsity that characterizes real-world signals, it is possible to recreate the acoustic environment. This is based on the notion that data acquisition and compression can be performed simultaneously. In contrast to such an approach, the traditional Nyquist and Shannon sampling theorem requires a large amount of data to be acquired. It reduces the amount of data further within the compression phase [4]. Hence, applying compressive sensing to the acquisition of acoustic signals rather than traditional sampling provides less sampled data. An example of acoustic sensing is the measurement of reverberation in a closed space to determine its intrusive influence on speech intelligibility.

However, some potential obstacles with sensing and reconstructing acoustic spaces accurately exist. One of the most important is reverberation that causes blurring of the acquired signals. Speech is one of the signals used in acoustic sensing technology. However, the primary use of speech is to communicate between people. Unfortunately, many factors impair the transmission of speech and make it less intelligible. This primarily concerns various types of noise, but in the case of indoors, one of the even more important problems is reverberation.

Speech intelligibility is often evaluated in noise and reverberation conditions. The so-called Lombard effect is a widely known phenomenon that is induced when the speaker unconsciously changes acoustic features of uttered speech in noise conditions. Even though this effect is known from 1911, there are many studies that have analyzed Lombard speech in the last two decades [5,6,7,8,9,10,11,12], especially as there are no indicators or measures that apply directly to assessing the quality of speech in such conditions [13,14,15]. In contrast, there are not too many works on the effect of reverberation on speech uttering and to what degree it affects speech intelligibility [16,17,18,19]. However, adjusting the rate of speech in reverberation conditions is similar to modifying the volume in Lombard speech. Indeed, in both cases, these adjustments are not a simple change in speech rate or volume increase. Both the behavioral contexts of vocalizations, as well as the resulting signal modifications, should be taken into account. In Lombard speech, effects such as the following are observed: increasing the level of sound energy, raising the fundamental frequency of the signal, shifting energy from lower frequency bands to higher frequency bands, increasing formants, the duration of vowels, the spectral tilt, etc. [8,10,20,21]. On the other hand, reverberation enforces persons to slow down their speech, but the differences in slowed speech over uttered word distribution are not uniform [17,18]. They depend not only on the speaker’s way of speaking, pronunciation, articulation, etc., but also on the type of masking that occurs in such conditions; here, it should be remembered that it concerns overlap masking in reverberant conditions [17]. In addition, it is of importance whether it involves people with normal hearing, hard-of-hearing people, or those with cochlear implants [16]. Another factor is related to the age of the listeners. The way to speak to children or older people differs from the everyday manner of talking. However, in this study, we limit the experiments to three factors, namely reverberation, rate of speaking, and speech intelligibility; otherwise, too many variables that depend on each other would be involved. Therefore, the experimental design aimed at controlling all measured factors. In future work, we plan to pursue threads related to the way of speaking and the group of people under reverberation conditions.

Reverberation affects speech in many ways. An extensive discussion of distortion caused by reverberation can be found in [22]. It shows that reverberation causes distortions mainly in the time domain. Reverberation fills in natural gaps between different parts of speech, blurs the temporal relationships between vowels and syllables, and prolongs noise fragments. It also affects the shape of formants. The authors of [23] similarly describe the impact of reverberation. They point out that the degradation of speech caused by reverberation is related to the phenomenon called overlap masking—parts of speech are masked by reverberant components of the preceding segment. To some extent, normal-hearing adults can understand speech under these conditions [24]. This is possible due to the redundant nature of the speech signal [22,25].

Unfortunately, the situation deteriorates significantly for those with hearing problems, including the aging population [26,27,28,29,30,31,32]. Harris and Swenson assessed speech intelligibility for people with and without sensorineural hearing impairment in three acoustic environments [33]. Mean speech recognition decreased with increasing reverberation time for each group. More importantly, the more severe the hearing impairment, the poorer was the test participants’ performance. Indeed, reverberation can also become a significant problem even for people with normal hearing and impede their ability to understand degraded speech. This occurs mainly in spaces with very large reverberation times, e.g., halls, atria, and multistory garages. Many reflective surfaces with very limited possibilities to absorb or diffuse the sound wave lead to a very rapid loss of intelligibility [34]. As a result, speech can be audible and heard but may not be comprehensible. 

Furthermore, perceived speech intelligibility may vary according to language. Interesting studies in this area have been conducted by Kitapci and Galbrun [35,36]. They were investigating the comparison of speech intelligibility in four languages: English, Polish, Arabic, and Mandarin. In [35], they focused on three acoustic environments (i.e., airport, hospital, and café). Three different acoustic conditions were chosen for each room type, with the following STI (speech transmission index [37]) values: 0.4, 0.5, and 0.6. The analysis of the perceived speech intelligibility results shows that English is the most sensitive to changes in STI values, and Polish is the least intelligible language. Arabic seems to be more resistant to deterioration of the acoustic conditions. The authors note, however, that these results are not entirely consistent with those they obtained in [36]. This concerns, for example, the Polish language. Nonetheless, the differences between the languages are evident.

The influence of language on the relationship between the actual speech intelligibility and the one estimated by STI has become the object of research of many scientists, e.g., [38,39]. Moreover, different languages and speech types, signal-to-noise ratios, and finally reverberant conditions can over- or underestimate actual speech intelligibility [18,38,40,41]. Liu et al. in [40] pointed out that this problem is especially true for larger spaces, with high reverberation times and small SNR (signal-to-noise ratio).

For many years, research has been carried out to find a way of processing speech to improve its intelligibility. Various methods can be found in the literature that are recommended to be used in reverberation environments. Cole et al. in [42] proposed the use of inverse impulse response of the room. Listening tests proved the effectiveness of this approach. Unfortunately, it is not always possible to use this method, e.g., in rooms with a larger volume, where the acoustic parameters change depending on the listener’s position. Reinhart and Souza in [27] tested the efficacy of a method based on varying the wide dynamic range compression (WDRC) release time. Subjects with sensorineural hearing loss participated in the study. It turned out that increasing the WDRC release time leads to improvements in the intelligibility of the thus processed speech. A different approach was adopted by Arai et al. [43]. They detected steady-state portions of speech and then reduced their power. In this way, it was possible to decrease the overlap masking caused by reverberation. The experiments (involving syllables in Japanese) showed that subjects performed better while listening to the processed speech. This method was then developed by Mzah et al. [23]. The main difference was the modification of the steady-state detection technique. The objective and subjective tests proved that the processed utterances (in French) were better recognized by subjects. The efficacy of the speech preprocessing method based on steady-state suppression led to its various modifications and improvements [44,45,46].

A certain development of this method is the algorithm proposed by Grosse and van der Par [47] comprising onset enhancement and overlap-masking reduction. The proposed method proved to be more effective than overlap-masking reduction even for rooms with a reverberation time of 3 s. The authors point out that adjusting the parameters of the algorithm to the specific properties of the room allows for improving its performance. The effectiveness of this method was confirmed by Bederna et al. also for rooms with reverberation time up to 4 s [48]. In the same conditions, the authors tested the AdaptDRC algorithm (originally designed to improve speech intelligibility in noisy environments), which proved to be effective even in the presence of reverberation. The AdaptDRC algorithm combines time- and frequency-dependent signal shaping with a time- and frequency-dependent dynamic range compression [49]. This method was tested in the Hurricane Challenge 2.0, which evaluated different algorithms designed to improve speech intelligibility [50]. The tests covered various conditions: three languages, three SNR values, and three reverberant conditions. It should be noted, however, that the simulated room was a cafeteria with a reverberation time of 0.8 s, and changes in reverberation were achieved by increasing the distance between source and receiver. A total of nine algorithms were tested in the Hurricane Challenge 2.0 [50], of which only three were described as “reverberation dependent.” AdaptDRC performed relatively poorly in this comparison for both German and Spanish. For English, the differences were not as significant. It was also evident that it performed better in more reverberant conditions. The other two reverberation-dependent algorithms (MS500 [51] and IISPA [52]) performed slightly worse. Of all the algorithms, ASE (the Automatic Sound Engineer) was the best in performance evaluation. ASE is based on dynamic compression performed in six bands [53]. The authors point out that the main feature of the method is that stimuli are processed independently from channel conditions. Certainly, its advantages also include relatively low computational complexity.

A relatively advanced method was proposed by Dong and Lee in [54]—it is a combination of the perceptual distortion measure–based speech enhancement (PDMSE) method and the fast inverse filtering (FIF) method. The results are promising, but the authors note that currently, this method does not allow for in-real-time operation.

In recent years, approaches using artificial neural network algorithms have been undertaken, such as [55,56]. It is possible that in the future, these methods will dominate the solutions implemented, but at present, this is a rather complicated approach. At present, it is not possible to operate this type of system in real time.

Methods based on slowing down the rate of speech can also be found in the literature, such as in [44]. The authors investigated the impact of speech-rate slowing algorithms and found that even simple slowing down without additional processing improves speech intelligibility for reverberation time of about 2 s. These observations were also confirmed by Arai et al. in [45]. The main difference was the use of slightly shorter reverberation times in the latter study (Table 1). Some forms of speech slowing were also proposed by Petkov and Stylianou in [57]. Their two-stage speech modification algorithm employs adaptive gain control and so-called time warping. 

A summary of methods designed to improve speech intelligibility under reverberant conditions is provided in Table 1.

Interesting observations on speech perception under different listening conditions, including the presence of reverberation, can be found in [58]. The results indicate that in reverberant conditions (RT = 3.5 s), the speech was assessed by subjects as faster than with filtering (or no processing). This provides another argument for using speech slowing down to improve intelligibility in spaces with high reverberation times.

The results of experiments using speech rate reduction provided the background for the study described in this article. Therefore, the aim is to determine how much, for a specific reverberation condition characterized by the STI, the rate of speech (RoS) should be reduced to achieve reasonable speech intelligibility. RoS is an important parameter characterizing a given utterance. It depends, among other things, on prosody, the language of the utterance, the individual characteristics of the speaker, the gender of the speaker, the type of speech (read/spontaneous speech), the age of the speaker, and the emotional state. Furthermore, RoS can be determined in real time—with the use of algorithms analyzing the time-frequency dependencies of individual parts of speech. Examples include analysis of the instantaneous loudness of the signal [59,60], analysis of the signal energy envelope [61], or vowel-detection algorithms [62,63,64].

Furthermore, discovering the optimal value of RoS that would allow almost any person (with normal hearing) to understand speech under specific acoustic conditions is another goal of this research. Moreover, processed speech should remain natural sounding (unnaturalness is one of the effects of slowing the speech rate excessively). Finally, acoustic sensing technology is built on hearing and hearing-processing principles, as human ears are the primary sensing “devices.” Therefore, if a human cannot understand speech in given conditions, then the same problem may certainly occur for technology.

Though the experiments performed by various researchers follow a long history of approaches to solving a problem of speech intelligibility in reverberation conditions, based on the literature review, we believe that our approach is original in the context of determining the relationship between the rate of speech and speech intelligibly in reverberation conditions based on the STI measure. We conceptualize a way to broadcast announcements (including emergency announcements) in reverberation environments, making them more intelligible by reducing the rate of speech (RoS) in real time. The protocol of the experiments focuses on determining the RoS conditioned by the reverberation time in relation to the measured STI values.

## 2. Materials and Methods

The experiments performed are based on the speech intelligibility tests for the Polish language developed by Ozimek et al. [65]. Since the objective of the study was to test sentence intelligibility, it was decided to use the modified Polish Matrix Test. The sentences have a fixed structure and are grammatically correct, although they have little contextual content. Each sentence consists of five words: name, verb, numeral, adjective, and noun. An example sentence looks like this: “Tomasz nosi pięć dobrych piłek” (Thomas carries five good balls). The individual words forming the utterances are selected at random. 

The words used to create the sentence material were read by a male speaker. The words were recorded in the form of ten sentences expressed in conversational speech. They contained all of the required words and took into account the syntactic structure of the tests. This way of recording the verbal material preserved the natural prosody of the spoken sentences. In the editing process, each word was cut out, and 200 ms of natural noise, being room noise, was included at its beginning and end. This was intended to allow consecutive words to be naturally combined into sentences. Sentence synthesis consisted of combining the corresponding words by merging areas of silence using a linear cross-fade. The average RoS of the recorded utterances was 6.48 vowels/s (standard deviation was 0.44 vowels/s).

**Figure 1:**
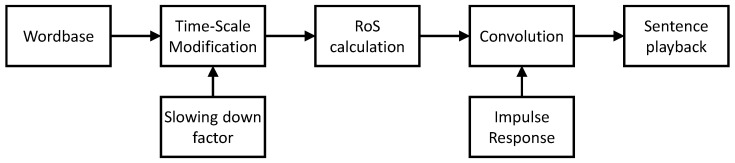
Flowchart of the sentence formation process.

The sentence thus created is then slowed down, and the RoS of the slowed utterance is calculated employing the length of the slowed utterance and the number of vowels in the sentence. The number of vowels is known a priori for each word in the sentence. In the next step, the slowed speech is convolved with the acquired impulse response of a given room. The resulting file is played back. The process of sentence formation is presented in Figure 1.

The subject’s task is to repeat the sentence and assess the rate of speech (appropriate or too fast). The supervisor verifies the correctness of the response. If the answer is incorrect, the factor of slowing down is increased by 20%. A single test consists of 13 trials; the first three trials are designed as a pre-test to familiarize the test subject with the sounds they need to recognize. The order of impulse responses for each subject was randomly assigned to reduce the possible influence of the listener’s learning of individual sounds. If the order was always the same, the results for the first impulse response tested might be worse than for the last impulse response case listened to, i.e., when the subject already knows the purpose of the test and what utterance to expect.

The tests were organized in two stages. The first stage used three impulse responses recorded at locations with low STI values (No. 1–3, STI range 0.34–0.45); the second stage used impulse responses recorded at sites with higher intelligibility (No. 4–6, STI range 0.53–0.76). Response No. 3 had the lowest STI value (of 0.34), while response No. 6 had the highest value (of 0.76).

The first stage results were crucial since the most significant improvement in intelligibility was expected for these conditions. The impulse responses were recorded in existing rooms—multistory parking garages of two local shopping malls. Brüel & Kjær measuring equipment (PULSE system with measuring microphone) was used to obtain impulse responses. Table 2 and Table 3 provide information on the impulse responses used and the acoustics of the locations in which they were collected.

An application in the MATLAB environment was developed for carrying out the tests. The core of the slowed speech method is the well-known SOLA (synchronous overlap-add) algorithm. This method was proposed by Roucos and Wilgus in [66] and is a modification of the OLA (overlap-add) method. The OLA method consists of two steps: analysis and synthesis. In the analysis step, frames of length *L* samples are taken from the input signal with a fixed time shift, *Ta*. During synthesis, the time shift length is changed and becomes *Ts*. Overlapping areas of neighboring frames are summed together using a cross-fade operation. The ratio of the synthesis and analysis step lengths determines the value of the scale factor according to the formula:

(1) $$\alpha =\frac{Ts}{Ta}.$$

Unfortunately, the synthesized signal is distorted due to the discontinuity of phase and amplitude in the summed frames. The authors of the SOLA method proposed to reduce these problems by analyzing the similarity of the signal in subsequent frames with the use of the cross-correlation function. To obtain the highest possible quality of the modified signal, the location of the maximum value of the correlation function is found. It determines the time offset that has to be added to the synthesis time shift to avoid time discontinuities.

There are also other modifications of the OLA method, such as WSOLA (waveform similarity overlap and add) [67], PSOLA (pitch synchronous overlap add) [68], and AOLA (adaptive overlap and add) [69]. However, based on the results of previous studies ([70,71]), we decided to choose SOLA due to its good resolution because it provides high-quality slowed speech and, at the same time, is not computationally demanding.

**Figure 2:**
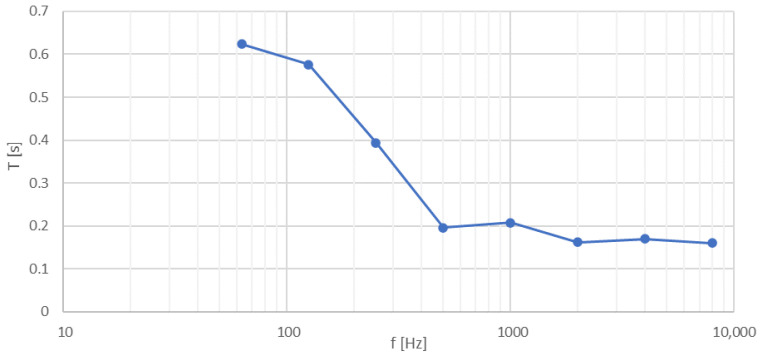
Reverberation time characteristics of the test room.

Listening tests were performed in an acoustically adapted room with a volume of 68 m^3^ and a floor area of 24 m^2^. The reverberation time characteristic of the room is shown in Figure 2.

Audio from a laptop computer running MATLAB was transmitted to an amplifier using an HDMI connection. The sound was played through seven NEXO PS8 full-range active loudspeakers. Each speaker played the same sound, which allowed the impression of sound surrounding the subject. During the test, the subject was positioned at the best listening spot (the so-called sweet spot). The sound level was set to obtain a comfortable volume for the subject.

A total of 15 participants took part in the listening tests (3 females, 12 males). Their ages ranged between 24 and 42 years. All participants had previous hearing tests that revealed no impairments.

## 3. Results

The obtained results could not be analyzed in a typical way—calculating the mean or median for the obtained values of the RoS parameter is not the solution to the problem. According to the objectives, the aim was to ensure intelligibility in the vast majority of cases (subjects). Hence, the obtained results for each impulse response were analyzed in the following way:

1. The maximum value of RoS for which the subject repeated the sentence correctly was found (i.e., the maximum rate at which the utterance was still intelligible).
2. The minimum value of RoS for which the person was not able to repeat the sentence correctly was found (i.e., the lowest rate at which the utterance was unintelligible).
3. The second-highest value of RoS for which the person repeated the sentences correctly was taken as the optimal value. In this way, random correct answers were eliminated.

**Figure 3:**
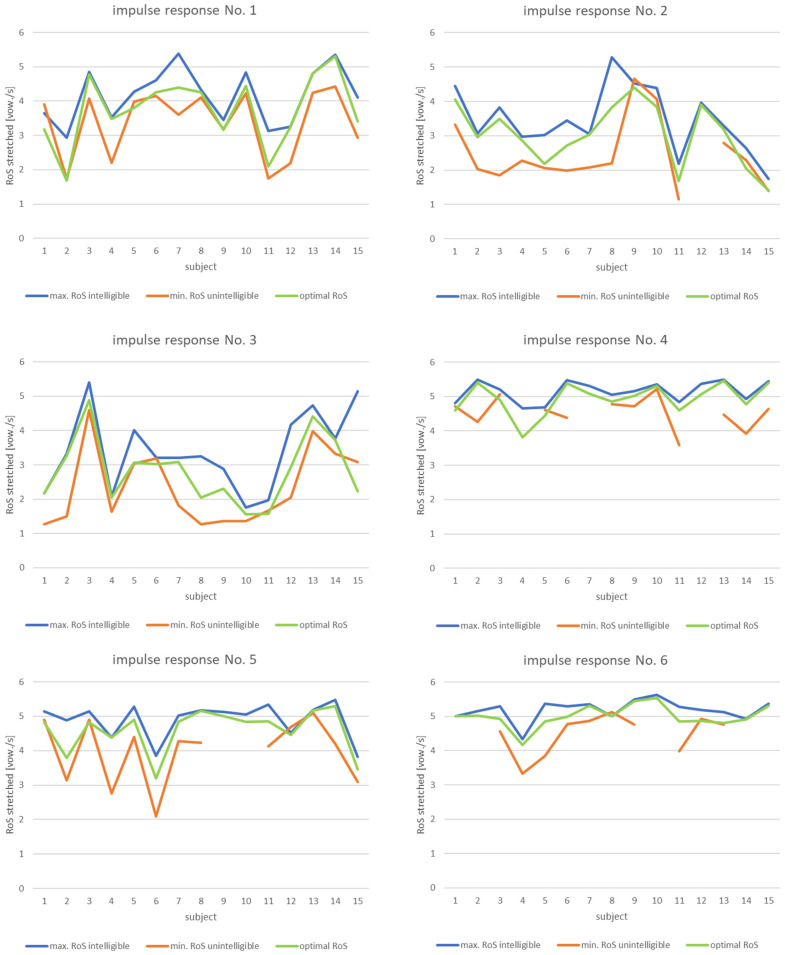
Results for obtained for particular impulse responses.

Figure 3 contains the results obtained for all responses. No value for the graph “min. RoS incomprehensible” means that the person had no problems understanding the utterance regardless of the speech rate.

To visualize the data presented in Figure 3, one can provide a plot of RoS as a function of STI. As there are multiple answers for each impulse response (associated with a unique value of STI measure), data can be presented in the form of a boxplot.

**Figure 4:**
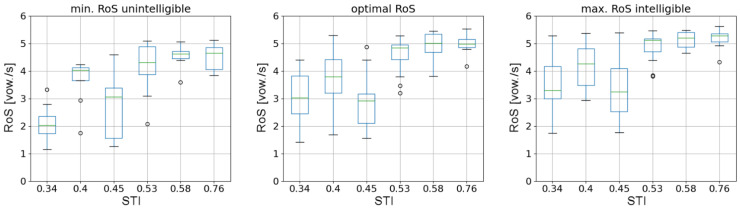
Boxplots depicting the RoS value as a function of STI associated with each of the six impulse responses considered in the study. The leftmost figure depicts RoS values associated with the lowest utterance tempo for which speech was already unintelligible, optimal RoS values for each STI are shown in the middle figure, the rightmost figure shows the maximum RoS value for which speech was intelligible for each STI value.

For each of the three parameters depicted in Figure 4, a rising trend can be observed. To prove statistical significance of such trends, a series of statistical tests was carried out using Python programming language. Testing procedures employed in the process can be found in the SciPy library (version 1.7.1) and StatsModels library (version 0.12.2). In the case of the measure depicting minimum RoS for which test sentences were already unintelligible, there were some participants for whom it was not possible to determine such values of RoS. Incomplete data vectors from those participants were omitted in the analysis. Therefore, for the optimal and maximum values of RoS, 15 data vectors from 15 participants were analyzed. For the minimum RoS, already unintelligible, only the remaining complete 8 data vectors were analyzed. For each of the statistical tests carried out in the testing process, a significance level of 0.05 was assumed.

The Levene statistical test was initially carried out for all three datasets. For the minimum RoS, the *p*-value was equal to 0.29 (test statistic of 1.28); for optimal RoS measure the *p*-value was less than 0.001 (test statistic of 4.18); finally, for maximum RoS the *p*-value was also less than 0.001 (test statistic of 5.74). This means that the assumption of the equality of variances imposed by the ANOVA statistical test holds only for the minimum RoS parameter. For two other parameters, the Levene test shows that variances are not equal for all groups presented in the boxplot. However, if the Shapiro–Wilk test for normality was carried out to check whether each group in the minimum RoS has Gaussian distribution, it turned out that one group was associated with a *p*-value of 0.02, which means, that finally even in the case of minimum RoS, ANOVA cannot be applied (due to the second assumptions of the ANOVA test). Therefore, for each parameter, the Kruskal–Wallis test, which is a nonparametric alternative to ANOVA, was performed.

For the minimum RoS, the *p*-value of the Kruskal–Wallis test was equal to less than 0.001 (test statistic of 25.87); for optimal RoS measure, the *p*-value was less than 0.001 (test statistic of 54.80); for maximum RoS, the *p*-value was also less than 0.001 (test statistic of 45.11). This means that for each considered parameter, at least one difference between two STI-related groups shown in Figure 4 was statistically significant. To identify such groups, a post hoc test had to be carried out. In the case of the Kruskal–Wallis test, a suitable post hoc test was Dunn’s test. Matrices of *p*-values are depicted in Table 4, Table 5 and Table 6.

For each matrix of *p*-values, a clear distinction of groups into two blocks can be seen—a block of low-STI RoS values (which are STIs of 0.34, 0.40, and 0.45), and a block of high-STI RoS values (STIs of 0.53, 0.58, and 0.76). Within those groups, there are no statistically significant differences for the optimal and maximal RoS values. This trend is less visible for the minimum RoS value, probably due to the smaller number of participants considered. The result of statistical tests allows for deriving a hypothesis that for increasing values of STI, there is a sudden change of acceptable RoS values, and this change is positioned between STIs of 0.45 and 0.53. Differences below and above this threshold were not found to be statistically significant. The trend was visible and statistically significant for all three considered RoS-related parameters.

According to the plots for the first three impulse responses (recorded in locations with poor speech intelligibility), it can be seen that the value of the optimal RoS for some of the subjects had minimum values below two vowels/s. For others, it was even significantly higher, reaching a value over four vowels/s. This shows how the ability to understand speech in reverberant conditions was an individual experience.

Based on the assumption that intelligibility should be provided in most cases, it seems advisable to consider the minimum values. Another important observation concerns the fact that, regardless of the STI value, the minimum optimal RoS values were very similar for all three responses. In determining the final RoS value, it should be considered that for a RoS below 2, the subjective quality (naturalness) of speech deteriorated significantly. Even the subjects pointed this out after the tests. Hence, the conclusion is that for locations with the poorest acoustic conditions, the optimal RoS value is 2. Thus, when using speech rate reduction algorithms, the factors of slowing down should be chosen so that the stretched speech reaches a value of two vowels per second.

The results for impulse responses No. 4–6 (recorded at locations with better acoustic conditions) show smaller differences in answers between subjects. It is also visible that lower values of the optimal RoS are obtained for impulse response No. 5, which is an expected result (lower STI than responses No. 4 and 6). In turn, the differences between the results for answers No. 4 and No. 6 were minimal, while the STI values differed significantly. Perhaps this was caused by differences between other acoustic parameters, such as EDT (early decay time) [72].

Examining the results for different participants and taking into account all impulse responses, it is difficult to discern some patterns. For example, participant No. 6 achieved the poorest result for impulse response No. 5, but in other cases did not differ from the other subjects. In reference to the determined optimal RoS values to the calculated average values for each response (STI values), it can be noted that the highest values were obtained by subjects No. 3 and 13. This means that they were able to understand faster utterances than the other subjects. In contrast, subjects No. 4 and 11 tended to require lower RoS values than the others, hence the lower optimal RoS values in their case. Such differences result from the individual abilities of subjects—their auditory systems adapt differently to acoustic conditions.

**Figure 5:**
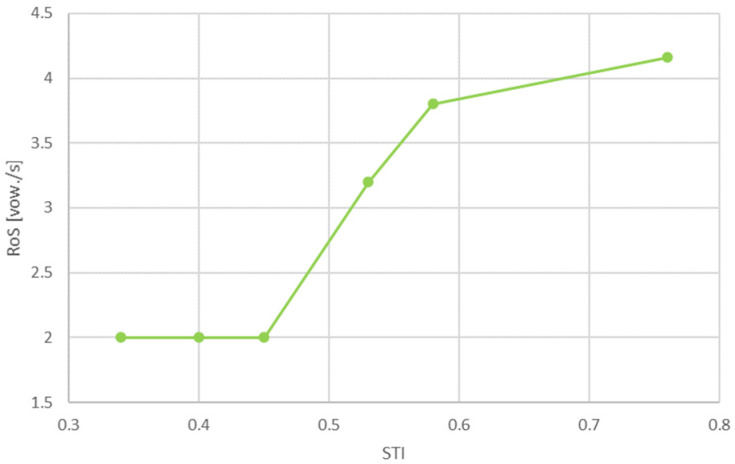
Developed curve showing RoS values as a function of the STI.

Considering the results obtained and the above discussion, a curve showing the optimal value of RoS for different STI values can be developed. It is presented in Figure 5.

**Figure 6:**
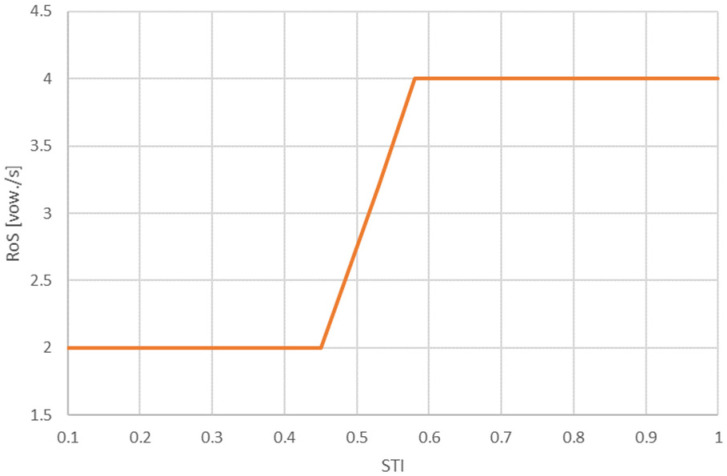
Simplified curve showing RoS values as a function of the STI.

It is possible to simplify this curve, improving its operation in a system using signal processors and sensors. The simplifications should not deteriorate speech intelligibility. Figure 6 shows the simplified curve. Table 7 provides numerical values for the significant points of both curves.

## 4. Discussion and Conclusions

The experiments performed revealed the maximum speech rate required to achieve sentence intelligibility for spaces with different acoustic conditions. The STI, one of the most critical objective parameters for assessing indoor speech intelligibility, was chosen as the reference parameter. Of course, other parameters could be selected, either directly related to reverberation time or EDT. In the literature, one can find numerous studies connecting the intelligibility issue with these two parameters [72,73,74]. It seems to us, however, that STI will be more applicable because it considers the entire transmission channel parameters, with noise and almost all types of distortions, and not just the acoustic conditions of the location [75].

Based on the results obtained, the curve was proposed to determine the required speech rate for different STI values. This curve, especially in its simplified version, is intended to support systems that perform automated speech preprocessing and, more specifically, to improve the intelligibility of speech by reducing its speed. Such a solution could be one of the components of public address systems used in indoor spaces, especially with a long reverberation time. Of course, modules responsible for estimating RoS and slowing down the speech signal will also be required. 

This is especially important in situations where the intelligibility of emergency announcements transmitted by multiple loudspeakers placed in a given space may be insufficient. This may affect the safety of the people in the area. Speech quality plays a smaller role in this case, i.e., high speech intelligibility is the most crucial factor. In turn, our research has shown that some of the distortion related to phase and amplitude discontinuities are masked by reverberation. This is an interesting observation that we will want to verify in future studies. 

Possible scenarios include the improvement of speech intelligibility in multistory garages, shopping malls, auditoriums, sports or exhibition halls, railroad stations, and airports. It is important to note that the proposed design allows for real-time speech processing, i.e., it is possible to process speech from a microphone. This feature of the system was taken into concern at the very beginning of the research work. Of course, in such a situation, it is necessary to ensure that the speaker does not hear his/her processed (slowed down speech), because it may make speaking difficult. This kind of limitation is not applicable when the system uses recorded speech (messages).

The next step of the work is planned to verify the effectiveness of the proposed curve in real-life conditions. We can expect some minor problems resulting mainly from the accuracy of the algorithms estimating the speech rate. As a result of such errors, the speech may be slowed down excessively, which not only will not improve intelligibility but instead may also make the speech sound unnatural. When the rate estimation algorithm overestimates the rate of speech, the processed speech will be too fast, and the improvement in intelligibility may not be sufficient.

Some limitations of the study should also be mentioned. First, the study was conducted only for one language (Polish). Based on the literature findings, it can be expected that for other languages, the curve of optimal RoS value will have a different shape. Another limitation is due to the relatively small group of subjects, none of whom had hearing impairments. One might expect that for older people and those with sensorineural disorders, it will be necessary to lower the optimal speech rate value for larger STI values. In contrast, it is not expected to introduce changes for lower STI values since speech with rates below two vowels/s sounds very unnatural. It also seems advisable to combine the time-scale modification algorithm with, e.g., steady-state suppression, which should improve intelligibility also for larger RoS. As a result, smaller slowing down factors might be used, which would also positively influence the quality of the processed speech.

Though a relationship between room impulse response, speaking rate, and speech intelligibility was established, there are several paths that may further be pursued. One of them concerns the more complex interior design, i.e., coupled rooms with complicated geometry. Such a case is more challenging to be solved analytically as each room may not only be characterized by different impulse responses, but the boundary conditions should be considered as well, especially in the case of the low-frequency sound field [76,77].

Moreover, in open spaces, additional factors should be taken into account, such as wind speed profile, vertical wind velocity gradients, the humidity of the air, temperature, background noise, and SNR (signal-to-noise ratio) in the case of noise. 

Summing up, slowing the speaking rate may provide speech enhancement in reverberant rooms. However, it may also be used as means of sensing room acoustics based on its impulse response and the speech convolved with it. Such a solution was proposed by Giri et al., who applied the sparse Bayesian learning approach for estimating room acoustics using reverberant recordings [78].

## Figures and Tables

**Table 1:** Summary of methods designed to improve speech intelligibility under reverberant conditions.

Citation	Method	Material	Subjects	Language	Reverberation Time
Cole et al. [42]	Inverse impulse response of the room	Phonetically balanced words	12 normal hearing	Australian English	-
Reinhart and Souza [27]	Varying wide dynamic range compression (WDRC) release time	160 low-context sentences	30 with sensorineural hearing loss	English	0.0, 0.5, 1.0, 2.0, 4.0 s
Arai et al. [43]	Steady-state suppression	Syllables	12 normal hearing	Japanese	1.1, 1.8 s
Mzah et al. [23]	Speech envelope modulation	VCV syllables	16 normal hearing	French	1.8 s
Nakata et al. [44]	Speech-rate slowing down and steady-state suppression	Nonsense consonant–vowel (CV) syllables embedded in a carrier phrase	24 normal hearing	Japanese	2.0, 2.8, 3.6 s
Arai et al. [45]	Slowing down and steady-state suppression	Nonsense consonant–vowel (CV) syllables embedded in a carrier phrase	25 normal hearing	Japanese	1.5, 2.0, 2.5 s
Arai [46]	Steady-state zero padding	Monosyllables embedded in a carrier phrase	31 normal hearing	Japanese	2.9, 3.3 s
Grosse and van der Par [47]	Overlap-masking reduction and onset enhancement	Sentences	8 normal hearing	German	0.7, 0.8, 1.2, 3 s
Bederna et al. [48]	AdaptDRC and onset enhancement	Sentences	17 normal hearing	German	1.08, 2.4, 4.14 s
Ngo et al. [51]	Modulation spectrum	Matrix sentences	Normal hearing, 62 German, 63 English, 62 Spanish	German, American English, and European Spanish	0.8 s (and three distances between source and receiver)
Schaedler [52]	Band-pass filtering, spectral modulation compression or expansion	Matrix sentences	Normal hearing, 62 German, 63 English, 62 Spanish	German, American English, and European Spanish	0.8 s (and three distances between source and receiver)
Chermaz and King [53]	Dynamic compression performed in six bands	Matrix sentences	Normal hearing, 62 German, 63 English, 62 Spanish	German, American English, and European Spanish	0.8 s (and three distances between source and receiver)
Dong and Lee [54]	Combination of the perceptual distortion measure–based speech enhancement (PDMSE) method and the fast inverse filtering (FIF) method	CVC syllables	18 normal hearing	English	0.08, 0.65, 1.39, 3.57 s
Chung et al. [55]	Deep convolutional neural network–based inverse filtering	TIMIT corpus (sentences)	Objective measures	English	0.5, 0.75, 1.0 s
Zhao and Wang [56]	Deep neural networks	WSJ0 corpus (news articles)	Objective measures	English	0.3 to 1.0 s, with 0.1 s increment
Petkov and Stylianou [57]	Adaptive gain control and time warping	Sentences	9 normal hearing	English	-

**Table 2:** Impulse responses used in the study and corresponding STI values (the lowest and the highest STI value are marked in bold).

Resp. No.	STI Value
1	0.45
2	**0.34**
3	0.40
4	0.58
5	0.53
6	**0.76**

**Table 3:** Reverberation times for the impulse responses collected in the study (bold denotes values corresponding to impulse responses with the lowest and highest STI values).

Resp. No./ f [Hz]	31.5	63	125	250	500	1000	2000	4000	8000	16,000
1	0.205	1.4	1.806	2.414	2.43	2.697	2.624	1.993	1.48	1.129
2	**-**	**0.406**	**2.048**	**2.223**	**2.624**	**2.52**	**2.623**	**2.026**	**1.507**	**1.057**
3	1.1	-	13.303	3.103	2.934	2.674	2.412	2.067	1.452	1.123
4	0.004	0.798	1.216	1.313	1.182	1.345	1.298	0.982	0.849	0.763
5	0.403	0.003	0.001	2.245	1.973	1.798	1.63	1.365	1.042	0.842
6	**0.246**	**0.006**	**4.537**	**1.427**	**1.718**	**1.549**	**0.642**	**0.725**	**0.932**	**0.774**

**Table 4:** Matrix of *p*-values obtained after carrying out Dunn’s post hoc test for the minimum RoS, for which utterances presented to the experiment participants were unintelligible. Statistically insignificant values are marked in bold.

STI Value	0.34	0.4	0.45	0.53	0.58	0.76
0.34		**0.063**	**0.401**	0.001	0.000	0.000
0.4	**0.063**		**0.309**	**0.180**	0.047	**0.056**
0.45	**0.401**	**0.309**		0.018	0.003	0.003
0.53	0.001	**0.180**	0.018		**0.520**	**0.568**
0.58	0.000	0.047	0.003	**0.520**		**0.943**
0.76	0.000	**0.056**	0.003	**0.568**	**0.943**	

**Table 5:** Matrix of *p*-values obtained after carrying out Dunn’s post hoc test for the optimal RoS value. Statistically insignificant values are marked with a gray background.

STI Value	0.34	0.4	0.45	0.53	0.58	0.76
0.34		**0.156**	**0.774**	0.000	0.000	0.000
0.4	**0.156**		**0.088**	0.033	0.001	0.000
0.45	**0.774**	**0.088**		0.000	0.000	0.000
0.53	0.000	0.033	0.000		**0.232**	**0.154**
0.58	0.000	0.001	0.000	**0.232**		**0.818**
0.76	0.000	0.000	0.000	**0.154**	**0.818**	

**Table 6:** Matrix of *p*-values obtained after carrying out Dunn’s post hoc test for the maximum intelligible RoS value. Statistically insignificant values are marked with a gray background.

STI Value	0.34	0.4	0.45	0.53	0.58	0.76
0.34		**0.152**	**0.878**	0.001	0.000	0.000
0.4	**0.152**		**0.201**	0.045	0.002	0.001
0.45	**0.878**	**0.201**		0.001	0.000	0.000
0.53	0.001	0.045	0.001		**0.258**	**0.225**
0.58	0.000	0.002	0.000	**0.258**		**0.936**
0.76	0.000	0.001	0.000	**0.225**	**0.936**	

**Table 7:** Proposed RoS values for both curves.

STI	0.1	0.34	0.40	0.45	0.53	0.58	0.76	1
RoS [vow./s]		2	2	2	3.2	3.8	4.1	
RoS simplified [vow./s]	2	2	2	2	3.2	4	4	4

## Data Availability

The data presented in this study are openly available in Most Danych at doi:10.34808/b0yr-cm79.
